# Approaches for quantifying antimicrobial consumption per animal species based on national sales data: a Swiss example, 2006 to 2013

**DOI:** 10.2807/1560-7917.ES.2017.22.6.30458

**Published:** 2017-02-09

**Authors:** Luís P Carmo, Gertraud Schüpbach-Regula, Cedric Müntener, Anne Chevance, Gérard Moulin, Ioannis Magouras

**Affiliations:** 1 Veterinary Public Health Institute, Vetsuisse, University of Bern, Switzerland; 2 Institut für Veterinärpharmakologie und –toxikologie, Vetsuisse, University of Zurich, Switzerland; 3 Anses - French Agency for Food, Environmental and Occupational Health & Safety, France

**Keywords:** antibiotic use, public health policy, surveillance, livestock, antimicrobial resistance

## Abstract

Antimicrobial use in animals is known to contribute to the global burden of antimicrobial resistance. Therefore, it is critical to monitor antimicrobial sales for livestock and pets. Despite the availability of veterinary antimicrobial sales data in most European countries, surveillance currently lacks consumption monitoring at the animal species level. In this study, alternative methods were investigated for stratifying antimicrobial sales per species using Swiss data (2006−2013). Three approaches were considered: (i) Equal Distribution (ED) allocated antimicrobial sales evenly across all species each product was licensed for; (ii) Biomass Distribution (BMD) stratified antimicrobial consumption, weighting the representativeness of each species' total biomass; and (iii) Longitudinal Study Extrapolation (LSE) assigned antimicrobial sales per species based on a field study describing prescription patterns in Switzerland. LSE is expected to provide the best estimates because it relies on field data. Given the Swiss example, BMD appears to be a reliable method when prescription data are not available, whereas ED seems to underestimate consumption in species with larger populations and higher treatment intensity. These methods represent a valuable tool for improving the monitoring systems of veterinary antimicrobial consumption across Europe.

## Introduction

Antimicrobial resistance has been gaining momentum as one of the most important topics within the public health sphere [1]. Part of the antimicrobial resistance burden for public health lies on the use of antimicrobials for veterinary purposes. Results from several studies have suggested that antimicrobial exposure in livestock is contributing to the emergence, selection and spread of antimicrobial resistant bacteria [2-4]. In addition, it is known that the use of antimicrobials in pets influences the resistance patterns found in those animals [5]. The subsequent spread of resistant bacteria from animals to humans can occur through multiple potential routes.

Monitoring systems in veterinary medicine can provide useful insights into temporal trends of antimicrobial consumption and ensure compliance with prudent usage practices, programmes or regulations. Furthermore, they can assist in identifying the most efficient interventions for optimising antimicrobial usage. When combined with antimicrobial resistance data, quantification of antimicrobial usage can be useful not only in identifying risk factors for the emergence of resistance, but also in describing temporal associations between antimicrobial usage and resistance [6,7]. Finally, monitoring systems can be a source of highly informative data for boosting research on the complex topic of emergence, selection and spread of antimicrobial resistance. Thus, monitoring antimicrobial consumption in livestock and companion animals is undoubtedly an important tool in the battle against antimicrobial resistance.

Research on the veterinary use of antimicrobials has focused on livestock species because their populations are larger and their antimicrobial consumption is higher than that of pet animals. Recognition of the importance of quantifying antimicrobial use in livestock emerged more than a decade ago [8] and the European Commission and the European Medicines Agency (EMA) have also emphasised the importance of monitoring antimicrobial use [9-11]. There is no binding European Union (EU) legislation with respect to the implementation of such monitoring programmes at national level and it is up to each country to define its strategy. 

In Switzerland, a non-EU country, the legal basis for sales data collection was defined in Article 35 of the Federal Ordinance on Veterinary Medicinal Products, enacted in September 2004 [12].

The European Surveillance of Veterinary Antimicrobial Consumption (ESVAC) project, initiated in 2010 by the EMA, has contributed considerably to the collection of standardised data on veterinary consumption in Europe [13]. ESVAC reports are published annually and are currently based on data provided by 26 countries, including Switzerland [14].

Prompted by the European Commission’s Action plan against the rising threats from Antimicrobial Resistance [10], ESVAC published guidance for data collection on antimicrobial consumption at the species level [15]. Furthermore, international guidelines such as the World Organisation for Animal Health’s Terrestrial Animal Health Code [7] and Integrated Surveillance of Antimicrobial Resistance: Guidance from a WHO Advisory Group [16], mention usage data at the species level as an important aspect that should be considered in monitoring systems. Data at the species and the production type level (such as dairy or beef cattle; broilers or laying hens; breeding, farrowing or fattening units) provide a better estimate of the antimicrobial exposure in each population and are therefore much more informative than overall sales data.

In mid-2000, Denmark implemented an automated system for nationwide collection of antimicrobial prescription data for production animals (pigs, poultry, cattle, sheep, goats, fish and mink) [6]. Systems providing data at the farm level can be used to identify high consumers and therefore implement benchmarking systems based on usage by individual farms or practitioners [17-19]. However, such systems can be very demanding in terms of resources and infrastructure [16] which might hinder their establishment.

In the absence of automated data collection schemes, alternatives need to be explored. ESVAC suggests that estimates of antimicrobial usage per species can be obtained through cross-sectional or longitudinal studies or based on data from marketing authorisation holders [15]. Some of these strategies have already been applied. In the Netherlands, longitudinal data on antimicrobial usage were collected from a sample of farms [20]. In France, antimicrobial consumption per animal species has been calculated based on estimates of marketing authorisation holders since 2009 [21].

In Switzerland, antimicrobial sales data have been obtained yearly at national level since 2004 by requesting the number of packages sold per product from the marketing authorisation holders [22]. However, this strategy does not enable quantification of antimicrobial consumption at the species level.

There is no standardised method for quantifying the distribution of antimicrobial sales per animal species. The choice of the method also depends on data availability. It is therefore of interest to compare different possible methods and observe how results vary.

The aim of this study was to propose and compare alternative methods for estimating the antimicrobial consumption in pet and livestock animal species or groups of species in Switzerland by combining sales data with (i) summary of product characteristics; (ii) summary of product characteristics and animal demographic data; (iii) prescription data from a longitudinal study.

## Methods

### Antimicrobial sales data and product information

Based on information obtained from marketing authorisation holders, the number of antimicrobial packages sold per product is converted into the corresponding amount of active ingredient. The results are published in the ARCH-Vet report (the official annual report on sales of antibiotics in veterinary medicine and antibiotic resistance monitoring of livestock in Switzerland) by the Federal Food Safety and Veterinary Office (FSVO) [23].

The FSVO granted the authors access to the detailed antimicrobial sales database. The models developed in this study were fed with sales data from the period 2006−2013. Results from these models are a proxy for antimicrobial consumption.

Antimicrobial products were categorised into two groups: monospecies products (authorised for a single species) and multispecies products (licensed for multiple species). This stratification was done by extracting from the Swiss Veterinary Drug Compendium data on the species each product is licensed for [24]. The following species or groups of related species (hereafter referred to as ‘species’) were considered: pigs, cattle, poultry, small ruminants (goats and sheep grouped together), horses and pets (cats and dogs grouped together). Poultry is roughly equivalent to the number of chickens because turkey or waterfowl production in Switzerland is negligible. It should also be noted that, in Switzerland, most horses are kept for leisure and only a small number enter the food chain.

Rabbits and fish were excluded as their population sizes are comparatively small, and therefore these groups are expected to represent a negligible contribution to the consumption of antimicrobials in Switzerland.

### Animal demographic data

The national total biomass of each species was calculated from 2006 to 2013 using the population correction unit (PCU) method. PCU is a technical unit of measurement. One PCU is equivalent to 1 kg of biomass of livestock and slaughtered animals [13]. For livestock, theoretical weights at the most likely time for treatment were based on ESVAC recommendations [15]. For cats and dogs we used 5 kg and 20 kg bodyweight, respectively, as these are accepted standards for drug regulatory agencies [25].

When possible, sources of demographic data used for the ESVAC report were consulted [26]. For pets, demographic data were collected from the Société pour l’alimentation des animaux familiers (Swiss Society for Pet Nutrition) [27,28]. For the years where no data were available (2009, 2011, 2013), the mean of the previous and the following year was used.

### Field data on antimicrobial prescription patterns

Regula et al. (2009) assessed the prescription patterns of veterinarians in Switzerland for the period 2004−2005 [29]. Eight veterinary practices, representing 1.5% of all veterinary clinics in Switzerland (with a total of 15 veterinarians), were selected based on the proportion of owners keeping livestock and the use of electronic databases for disease and prescription records. Cattle, pigs, sheep, goats, horses, dogs and cats were included in this study. The proportion of animals at risk of being treated in the field study relative to the total number of animals in the country varied across the different animal species. To take this into account, the total amount of active ingredient prescribed was divided by the percentage of animals of each species at risk of being treated. The number of animals at risk of being treated during the field study were calculated as follows: (i) for horses, the number of owners in the practice records was used as a proxy for the number of animals (ii) for pets, the number of owners in the practice records was multiplied by the mean number of pets per household in Switzerland [27,28]; (iii) for pigs and cattle, veterinarians enrolled in the study provided estimates of the number of animals on the farms they visited; (iv) the number of small ruminants at risk of being treated was calculated based on the number of cattle at risk of being treated. We assumed that the ratio of cattle to small ruminants in the field study was the same as at the national level [26].

Field data were used to estimate the distribution of antimicrobial consumption by different species. These estimates were used to calculate mode values of Program Evaluation and Review Technique (PERT) distributions used in the Longitudinal Study Extrapolation (LSE) model described in detail below.

### Distribution of antimicrobial sales per species

Three different methods were used to extrapolate antimicrobial usage per species from sales data: Equal Distribution (ED), Biomass Distribution (BMD) and Longitudinal Study Extrapolation (LSE). Each method was exemplified using Swiss data, allowing for the calculation of estimates of antimicrobial consumption for several animal species from 2006 to 2013. Data analyses were performed using R statistical software [30].

Consumption estimates are presented in mg per PCU when referring to total national consumption and in mg per kg of biomass when describing the consumption by specific animal species.

### Equal Distribution 

ED assumed that antimicrobial consumption was equal for each species a product was licensed for. Thus, the amount of antimicrobial product used by a species in a given year was calculated as follows:

$C_{{p_{a},spec}_{a}{,y}_{a}}=\;\frac{S_{y_{a}}}{\sum {spec}_{n}}$

C: Consumption estimate; p_a_: a given product; y_a_: a given year; spec_a_: a given species for which a product is licensed; spec_n_: all the species for which a product is licensed; S: amount of product in sales.

The model was developed on a product basis. Calculated amounts of active ingredient belonging to the same antimicrobial class were summed for each year and animal species.

### Biomass Distribution 

In this method, the amount of product sold (in 2006−2013) was distributed proportionally to the relative importance of a species’ total biomass at a national level. The analysis was done for each product individually, taking into account the animal species the product is licensed for and the corresponding annual biomass values. The calculation for every product was performed as follows:

$C_{{p_{a},spec}_{a}{,y}_{a}}=S_{y_{a}}*\;\frac{{BM}_{{spec}_{a}{,y}_{a}}}{\sum {BM}_{{spec}_{n}{,y}_{a}}}$

C: Consumption estimate; p_a_: a given product; y_a_: a given year; spec_a_: a given species a product is licensed for; spec_n_: all the species a product is licensed for; S: amount of product in sales; BM: biomass.

Finally, the results were summed up for every combination of animal species, antimicrobial class and year.

### Longitudinal Study Extrapolation 

In this approach, estimates of the antimicrobial sales repartition per species (i.e. the amount of antimicrobials sold for use by each species) were derived from a Monte Carlo simulation, using PERT distributions to model the uncertainty of the data derived from the longitudinal study. This type of beta distribution is generated from three values: minimum (Min), mode and maximum (Max). PERT distributions were created for every combination of antimicrobial class, year and animal species. The values in these distributions ranged from 0 to 1 and represented proportions of the total amount of sales for the respective antimicrobial class in a given year.

Min and Max were calculated by combining sales data with information from the Swiss Veterinary Drug Compendium [24]. Min was estimated by summing the amounts of monospecies products sold for each combination of antimicrobial class, animal species and year. Max was calculated as the sum of the amounts sold of all the products (monospecies and multispecies products) of a certain antimicrobial class licensed for a specific species, in a specific year.

Both Min and Max values were converted into a proportion of the total amount of antimicrobial for the same combination of antimicrobial class and year. In summary, Min and Max were calculated as follows:

${Min}_{{spec}_{a}{,y}_{a},{AMC}_{a}}=\;\frac{\sum {Mono}_{{spec}_{a}{,y}_{a},{AMC}_{a}}}{S_{y_{a},{AMC}_{a}}}$

${Max}_{{spec}_{a}{,y}_{a},{AMC}_{a}}=\;\frac{\sum {Mono}_{{spec}_{a}{,y}_{a},{AMC}_{a}}+\;\sum {Multi}_{{spec}_{a}{,y}_{a},{AMC}_{a}}}{S_{y_{a},{AMC}_{a}}}$

Min: minimum of the PERT distribution; Max: maximum of the PERT distribution; Mono: monospecies products; Multi: multispecies products; spec_a_: a given species a product is licensed for; y_a_: a given year; AMCa: a given antimicrobial class; S: amount of product in sales.

Mode values of the PERT distributions were based on data from the field study on antimicrobial prescription patterns in Switzerland [29]. Specifically, the total amount of active ingredient from each antimicrobial class prescribed for each species was divided by the total amount of authorised products for that same species and antimicrobial class. This value was then used to estimate a mode value between the Min and the Max. The mode for each combination of species, antimicrobial class and year was calculated as follows:

${Mode}_{{spec}_{a}{,y}_{a},{AMC}_{a}}=\left(\frac{{F_{Mono}}_{{spec}_{a},{AMC}_{a}}+\;{F_{Multi}}_{{spec}_{a},{AMC}_{a}}}{{F_{Mono}}_{{spec}_{n},{AMC}_{a}}+\;{F_{Multi}}_{{spec}_{n},{AMC}_{a}}}\right)*({Max}_{{spec}_{a}{,y}_{a},{AMC}_{a}}-\;{Min}_{{spec}_{a}{,y}_{a},{AMC}_{a}})+\;{Min}_{{spec}_{a}{,y}_{a},{AMC}_{a}}$

Mode: mode of the PERT distribution; F: amount of antimicrobial from the field study; Min: minimum of the PERT distribution; Max: maximum of the PERT distribution; Mono: monospecies products; Multi: multispecies products; spec_a_: a given species a product is licensed for; y_a_: a given year; AMCa: a given antimicrobial class; spec_n:_: all the species a product is licensed for.

Poultry was not included in the field study, and therefore mode values for the PERT distributions of this species group were calculated as the mean value of the Min and Max for each year.

The mode values were standardised so that they added up to 1 for each combination of antimicrobial class and year. For this, the mode values were recalculated proportionally to their species distribution in the longitudinal study.

The simulation model was then run 10,000 times, using the R package ‘mc2d’ [31], and the mean of the results of each iteration was calculated. This result represented the proportion of the total sales of a certain antimicrobial class in a given year that was sold for consumption by a given animal species. Due to the stochasticity of the model and the skewness of some distributions (particularly when the mode value was close to the Min or Max), the sum of the repartition per species was often different from 100% for each year/antimicrobial class combination. For that reason, the repartition values were standardised proportionally to each species estimates. The 95% credibility intervals for each estimate were calculated using the R package ‘stats’ [30]. Finally, these values were used to estimate, for each antimicrobial class and year, the amount of antibiotics sold for use by each of the animal species.

For the three models, results are presented at an antimicrobial class level as the total amount of antimicrobials sold in kg and as mg of active ingredient sold per kg biomass.

Due to confidentiality reasons, no results disclosing the sales of individual products or marketing authorisation holders can be presented. Thus, sales at an antimicrobial class level are not shown for some species.

## Results

### Descriptive statistics: veterinary antimicrobial sales data from Switzerland, 2006–2013

From 2006 to 2008 there was an increase in the sale of veterinary antimicrobial products from 67,423 kg of active ingredient to 72,300 kg. Starting in 2008, a steady decrease in sales was observed, resulting in a total reduction of 26.2% by 2013.

The amount of monospecies products sold throughout the study period ranged from 24.9% (2011) to 30.1% (2006) of the total amount of antimicrobial product sold. Multispecies products authorised for two species represented 51.6–56.4% of the total amount of antimicrobial sold in each of the 8 years considered.

When the total sales were converted into mg per PCU, it was observed that variations in animal demographics did not influence the antimicrobial consumption pattern. Sales per PCU peaked in 2008 (87.9 mg/PCU); in 2013, the sale of veterinary antimicrobial products reached a minimum of 64.5 mg/PCU (Table 1).

**Table 1:** Sales (mg per population correction unit) for different antimicrobial classes in Switzerland, 2006­–2013

Veterinary antimicrobial sales in mg per PCU								
	**2006**	**2007**	**2008**	**2009**	**2010**	**2011**	**2012**	**2013**
Aminoglycosides	4.6	4.6	4.5	4.3	3.9	4.0	3.9	3.8
Cephalosporins	0.6	0.6	0.6	0.7	0.7	0.7	0.7	0.6
Fluoroquinolones	0.4	0.5	0.5	0.5	0.5	0.5	0.4	0.5
Macrolides	4.4	4.9	5.2	4.9	4.6	4.2	4.0	3.8
Penicillins	15.9	15.9	16.6	15.8	16.1	16.4	15.9	15.8
Polymyxins	2.3	2.0	1.9	1.9	1.8	1.7	1.3	1.0
Sulfonamides/trimethroprim	35.8	38.2	37.7	35.1	32.9	29.6	27.6	24.3
Tetracycline	18.5	20.5	20.3	18.8	17.7	16.5	14.5	14.1
Others	0.4	0.6	0.5	0.5	0.5	0.9	0.7	0.7
Total	82.9	87.8	87.9	82.5	78.6	74.5	68.7	64.5

PCU: population correction unit.

‘Others’ includes amphenicols, quinolones (other than fluoroquinolones), lincosamides, pleuromutilins

Sulfonamides, tetracyclines and penicillins were the antimicrobial classes sold the most throughout the years. Their contribution to the total sales ranged from 81.7% to 82.3% of the total mg per PCU. In parallel, sulfonamides and tetracyclines were the antimicrobial classes that contributed most to the observed decrease in antimicrobial consumption from 2008 to 2013, with decreases of 12.5 and 6.3 mg per PCU, respectively.

### Equal Distribution

**Figure 1:**
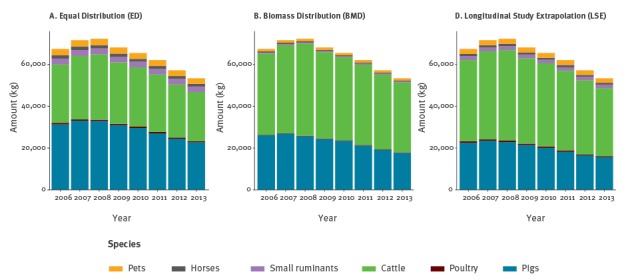
Distribution of total antimicrobial sales per species according to the three different approaches. Switzerland, 2006–2013

ED estimated that most of the antimicrobials (min−max, 2006−2013) were sold for use in pigs (42.6−46.4%) and cattle (41.4−44.1%). Over the years, a decrease in the sale of antimicrobials for pigs was observed. However, the proportion of antimicrobials sold for cattle increased, despite a reduction in the total amount of antimicrobials sold for this species (Figure 1).

Equal Distribution (ED): equal sales repartition by the species the products are licensed for.

Biomass Distribution (BMD): sales repartition based on the relative total biomass of the species in the country.

Longitudinal Study Extrapolation (LSE): sales repartition based on results from a previous field study. Pets are defined as cats and dogs, while small ruminants include goats and sheep. Horses are included as a leisure/sports species.

The estimates for poultry revealed a negligible contribution to the overall sales (0.9−1.1%).

When using mg of antimicrobial per kg biomass (mg/BM) as a consumption metric, pets (145.4−179.5 mg/BM) and pigs (110.0−160.0 mg/BM) were the species that seemed to be under the greatest antimicrobial pressure. Antimicrobials for cattle ranged from 48.8 to 64.5 mg/BM (Table 2).

**Table 2:** Antimicrobial consumption (mg per kg of biomass) for different animal species according to the three approaches in Switzerland, 2006–2013

Antimicrobial consumption in mg per kg of biomass																		
Year	Pigs			Poultry			Cattle			Small ruminants			Horses			Pets		
	ED	BMD	LSE	ED	BMD	LSE	ED	BMD	LSE	ED	BMD	LSE	ED	BMD	LSE	ED	BMD	LSE
2006	142.6	118.7	102.5 (54.9−178.6)	20.7	6.2	21.0 (6.9−34.9)	58.7	81.6	81.6 (47.1−103.3)	69.6	14.1	44.9 (2.1−130.5)	70.6	25.5	48.4 (15.7−116.0)	178.1	68.7	142.1 (58.7−300.7)
2007	153.6	124.6	109.1 (56.7−192.3)	13.4	5.1	13.9 (4.8−22.6)	64.2	89.1	89.1 (51.3−113.2)	72.0	14.4	45.3 (2.0−133.2)	72.8	27.7	49.3 (17.0−115.8)	174.6	70.9	139.0 (59.3−291.5)
2008	160.0	124.4	111.0 (54.0−201.3)	12.6	5.2	13.9 (5.0−22.6)	64.5	90.9	89.4 (50.8−113.7)	71.4	14.4	45.9 (2.2−133.9)	66.3	25.8	47.2 (16.4−110.1)	179.5	70.8	145.3 (60.9−302.5)
2009	147.5	115.9	102.3 (49.8−186.4)	12.4	5.1	12.7 (4.4−20.7)	60.6	85.2	84.3 (48.0−107.2)	70.9	13.8	44.9 (2.1−130.5)	61.9	21.8	42.9 (14.3−103.1)	175.1	70.7	141.9 (60.9−294.3)
2010	135.0	107.1	91.6 (42.1−170.2)	12.6	5.2	12.8 (4.5−21.2)	58.7	81.9	82.2 (46.0−104.7)	70.4	13.2	45.4 (2.1−132.5)	60.1	23.2	40.8 (15.1−94.3)	178.0	69.4	144.9 (61.0−304.6)
2011	124.6	97.8	84.0 (36.6−160.3)	12.9	5.6	12.2 (4.3−21.3)	56.8	79.4	78.7 (44.7−101.0)	70.6	13.2	45.0 (2.1−131.7)	62.3	24.2	49.3 (24.0−102.7)	168.0	67.0	137.3 (59.2−281.7)
2012	115.9	90.3	78.0 (33.7−149.4)	10.1	4.6	9.0 (3.2−15.4)	52.3	73.9	72.9 (42.2−92.4)	72.4	13.4	46.5 (2.1−134.6)	60.5	22.5	40.5 (13.3−97.3)	156.9	60.6	126.7 (52.6−263.9)
2013	111.0	86.3	76.4 (34.0−143.8)	8.8	4.3	8.2 (2.8−14.3)	48.8	69.3	67.4 (38.7−85.4)	71.9	13.3	45.9 (2.0−133.7)	54.3	19.6	36.6 (10.9−90.5)	145.4	57.3	117.3 (47.1−248.0)

BMD: biomass distribution; ED: equal distribution; LSE: longitudinal study extrapolation

Results for the three approaches:

ED: equal sales repartition by the species the products are licensed for.

BMD: sales repartition based on the relative total biomass of the species in the country.

LSE: sales repartition based on results from a previous field study) presented in mg per kg biomass. Results from LSE are presented as: mean (minimum 95% credibility interval – maximum 95% credibility interval). Goats and sheep were grouped together as ‘small ruminants’; cats and dogs were grouped together as ‘pets’. The species ‘horses’ is mainly represented by leisure animals

### Biomass Distribution 

Estimates from this model highlighted cattle as the species for which most antimicrobials were sold, with an increase in percentage from 57.6% in 2006 to 62.7% in 2013. Despite this result, the total amount of antimicrobials sold for cattle decreased from 38,809 kg to 33,446 kg in the same period. For pigs, percentages ranged from 38.7% in 2006 to 33.1% in 2013. For other species, the repartition estimates varied as follows: pets 1.7−1.9%; small ruminants 0.8−0.9%; horses 0.8−0.9%; poultry 0.3−0.9%.

Using mg/BM as a consumption metric, the values for the three main livestock species were between 86.3−124.4 mg/BM for swine, 69.3−90.9 mg/BM for cattle and 4.3−6.2 mg/BM for poultry (Table 2).

### Longitudinal Study Extrapolation 

With the exception of small ruminants (for which antimicrobial sales were relatively stable throughout the years), the LSE model calculated a reduction in antimicrobial consumption over time for all species. Total consumption of antimicrobials by cattle as a percentage of consumption by all species was lowest in 2006, at 57.6% (33.2−72.8%) (mean (minimum of the 95% credibility interval–maximum of the 95% credibility interval)) and highest in 2012, at 61.8% (35.7−78.3%). The consumption of antimicrobials by pigs went in the opposite direction, with a minimum of 28.8% (12.4−55.1%) in 2012 and a maximum of 33.4% (17.9−58.1%) in 2006.

**Figure 2:**
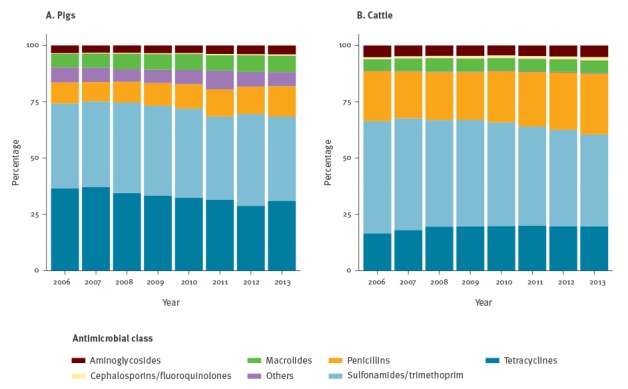
Relative distribution of the consumption of different antimicrobial classes for pigs and cattle, Switzerland, 2006–2013

Despite some differences in terms of the relative proportion of consumption of different antimicrobial classes (Figure 2), estimates for pigs and cattle decreased over time in terms of mg/BM: the estimated consumption by cattle dropped from 81.6 mg/BM (47.1−103.3 mg/BM) in 2006 to 67.4 mg/BM (38.7−85.4 mg/BM) in 2013; for pigs, consumption estimates went down from 102.5 mg/BM (54.9−178.6 mg/BM) to 76.4 mg/BM (34.0−143.8 mg/BM) in the same time period.

The estimates were obtained through the Longitudinal Study Extrapolation (LSE) approach: sales repartition based on results from a previous field study. ‘Others’ includes amphenicols, quinolones (other than fluoroquinolones), lincosamides, pleuromutilins and polymixins.

**Figure 3:**
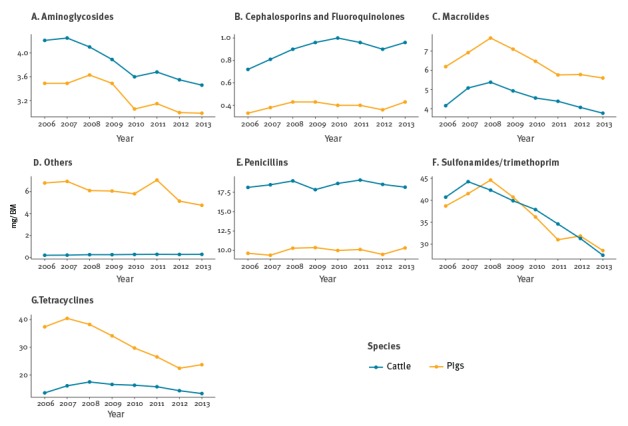
Estimated antimicrobial consumption of different antimicrobial classes for pigs and cattle, Switzerland, 2006–2013

For cattle, sulfonamides were the antimicrobial class that contributed the most to this decrease; for pigs, tetracyclines and sulfonamides were the classes for which consumption reduced the most (Figure 3).

Results are presented in mg per kg of biomass (mg/BM). The estimates were obtained through the Longitudinal Study Extrapolation approach (LSE): sales repartition based on results from a previous field study. ‘Others’ includes amphenicols, quinolones (other than fluoroquinolones), lincosamides, pleuromutilins and polymixins.

## Discussion

We compared different methods for stratifying antimicrobial sales data per animal species in Switzerland. This research follows the premise of the ESVAC project regarding the need to develop quantification methods of antimicrobial consumption at the species level [15].

The observed decrease of 26.2% in antimicrobial sales from 2008 to 2013 is most likely related to several concomitant reasons. Increased awareness by farmers and veterinarians about the issue of antimicrobial usage and resistance, due to several educational programmes by the FSVO, including the StAR programme [32], might have played a role in this decline. Viral diseases can lead to the use of antimicrobials to treat secondary infections [33,34]. For this reason, the implementation of the Bovine Viral Diarrhoea eradication programme and the commercialisation of Porcine Circovirus-2 vaccines might also have contributed to this reduction.

With regards to the results of the models, ED seemed to overestimate antimicrobial consumption for all species except cattle. ED does not consider variation in the levels of consumption by different species and does not take into account animal demographics, which might explain this overestimation. Indeed, it is likely that differences exist in the species repartition for multispecies products, especially in those shared between livestock and pet animals. Moreover, in Switzerland, the total cattle biomass was higher than for other species [35], largely due to the large number of dairy cattle. Given that this method did not take into account the existing number of animals in the country, it probably underestimated the usage for species with a higher total biomass and overestimated the usage for species with a lower total biomass.

The differences in the species total biomass values substantially influenced the BMD estimates, as biomass is the main driver for the repartition of sales data when using this approach. It was therefore not surprising that estimates of antimicrobial consumption by cattle were higher than those from ED. Concomitantly, extrapolated consumption by species with a lower biomass but high treatment intensity might have been underestimated.

For cattle and pigs, estimates from BMD and LSE were similar throughout the study period. The maximum difference between the two approaches was 16.2 mg/BM for pigs and 1.9 mg/BM for cattle. For the other species, LSE estimates tended to lie between ED and BMD results. The BMD approach seems to be a useful method when field data are not available. Nevertheless, extrapolation of this method to other countries should be done with care, as it is highly dependent on animal demographics.

The LSE method calculated a steady decrease in antimicrobial consumption throughout the studied years for most of the species. Small ruminants were an exception and were associated with a minor increase over the study period, from 44.9 mg/BM (2.1−130.5 mg/BM) to 45.9 mg/BM (2.0−133.7 mg/BM). Nevertheless, it should be highlighted that the uncertainty around these estimates is large.

In poultry, a steep decrease in the estimates of antimicrobial consumption (from 21.0 mg/BM (6.9 − 34.9 mg/BM) to 13.9 mg/BM (4.8 − 22.6 mg/BM)) was observed from 2006 to 2007. This change might be a model artefact and not a true reduction in antimicrobial consumption. As field data were unavailable for poultry, mode values of PERT distributions might not have been very accurate, especially for those antimicrobial classes where the difference between the Min and the Max was more accentuated. In those cases, it is likely that the mode used to represent poultry’s antimicrobial consumption was overestimated. In addition, this steep decrease might be partly related to the discontinuation of some products licensed for poultry between 2006 and 2008.

Pigs showed the largest decrease in antimicrobial consumption. We estimated that the pig producing industry had a particularly large antimicrobial consumption in the beginning of the study, and thus more opportunities to reduce usage were available, which might partially explain this steep decrease. Furthermore, the use of vaccines against Porcine Circovirus-2 and porcine proliferative enteritis (*Lawsonia intracellularis*) might also have played a role in the reduction of antimicrobial sales for use in pigs.

The wide use of Porcine Circovirus-2 vaccination in Switzerland might be associated with a lower prevalence of respiratory disease [36]. Tetracyclines are the main class used to treat respiratory disease in pigs [37]. We investigated whether LSE was able to capture this decline in specific antimicrobial classes. Indeed, tetracycline consumption showed a decline by 38.0% in pigs (larger than the decline for any other species) between 2008 and 2013.

On an antimicrobial class level, differences were observed between the classes used to treat pigs and cattle. Despite the general decrease in the usage of most antimicrobial classes, a slight increase in the consumption of cephalosporins and fluoroquinolones was estimated for cattle and pigs.

One of the most relevant benefits of having antimicrobial consumption estimates at the species level relates to the possibility of analysing them together with the resistance patterns from the national monitoring system. In Switzerland, indicator (*Escherichiacoli* and *Enterococcus* spp.) and zoonotic (*Salmonella* spp. and *Campylobacter* spp.) isolates from the three main livestock species (cattle, pigs and poultry) are collected every year. Regarding zoonotic bacteria, a general decrease in resistance was observed in Salmonella throughout the study period. This is in line with the reduction in antimicrobial consumption in the same period. For *Campylobacter jejuni* collected from poultry, a rise in the prevalence of ciprofloxacin resistant isolates was observed, from 12.0% in 2006 to 41.4% in 2013. In the same period, an increase in the consumption of fluoroquinolones was observed. This is of particular relevance for public health given that fluoroquinolones are the treatment of choice for severe Campylobacter infections. With respect to *Campylobacter coli* from pigs, a fairly stable level of resistant isolates was found [38]; the level of antimicrobial consumption did not seem to influence the resistance pattern observed.

Concerning the indicator bacteria, it is interesting to note that an increase of ciprofloxacin-resistant *E.coli* isolates was observed for poultry, which was more pronounced than that for other species. On the other hand, streptomycin resistance in *E. coli* was higher for cattle and pigs when compared with broilers. This might be related to the lack of licensed aminoglycoside products for use in Swiss poultry production [38].

It is important to highlight that when assessing temporal associations between antimicrobial usage data and resistance, other factors need to be taken into account, such as cross- and co-resistance, as well as the emergence and selection of specific clones. A comprehensive analysis of these temporal patterns and the effect of antimicrobial consumption on the resistance of animal isolates should be performed.

Although it is not possible to validate the models, we are convinced that the LSE approach provided the best estimates. In this approach, input data for the model are derived from a longitudinal field study. These data are closer to the actual usage of antimicrobials than sales data and are therefore more likely to reflect reality. Nonetheless, the LSE method also presents some potential bias. In the first place, the field data that fed the model were from the period 2004−2005. Consumption patterns may have changed since then. However, product repartition values are not expected to vary greatly from year to year. In Switzerland, marketing authorisation holders update their repartition estimates every 5 years. These estimates are used in the Periodic Safety Update Reports (PSURs) for calculating the incidence of adverse reactions. Nonetheless, we recommend performing field studies more frequently when applying this method to yearly monitoring. Another uncertainty might arise from the number of animals at risk of being treated in the field study, which was estimated from the participating veterinarians or calculated based on the number of farms/owners. This may have introduced some bias into the extrapolation of the field study results to a national level. When applying this method, it is advisable to have accurate estimates of the number of animals at risk of being treated in the field study. In addition to the methods presented, data for antimicrobial sales repartition per species might be obtained by asking the marketing authorisation holders [21]. It has not yet been possible to apply this valuable approach in Switzerland due to data limitations. Likewise, repartition estimates from PSURs can also provide a basis for sales stratification.

We presented three methods for extrapolating antimicrobial consumption per animal species from sales data. These approaches could be of use for countries which have not implemented detailed monitoring systems and which base their schemes on overall sales data. The best model choice in a given situation will depend on data availability. Results must always be interpreted in the light of data availability and country characteristics, and the limitations of each model must be considered. We shall also highlight that having consumption data per species enables the calculation of treatment incidence metrics, which better describe exposure to antimicrobials than mg per PCU. The LSE approach might also be of relevance for monitoring systems that rely on compliance of the people prescribing and administering antimicrobials to animals. In cases of imperfect compliance, a model that repartitions total sales data per species allows comparison of recorded vs expected amounts used. This might be very useful for targeted interventions to improve data quality of the monitoring system.
